# Standing and Lying Ni(OH)_2_ Nanosheets on Multilayer Graphene for High-Performance Supercapacitors

**DOI:** 10.3390/nano11071662

**Published:** 2021-06-24

**Authors:** Junming Xu, Mengxia Tang, Zhengming Hu, Xiaoping Hu, Tao Zhou, Kaixin Song, Jun Wu, Jipeng Cheng

**Affiliations:** 1 College of Electronic Information, Hangzhou Dianzi University, Hangzhou 310018, China; tmxemail@163.com (M.T.); hzm1994@hdu.edu.cn (Z.H.); huxp@hdu.edu.cn (X.H.); zhou.tao@hdu.edu.cn (T.Z.); kxsong@hdu.edu.cn (K.S.); wujun@hdu.edu.cn (J.W.); 2 School of Materials Science & Engineering, Zhejiang University, Hangzhou 310027, China; 3 School of Physics and Microelectronics, Zhengzhou University, Zhengzhou 450052, China

**Keywords:** Ni(OH)_2_ nanosheet, multilayer graphene, supercapacitor, molecular connection, Ni(OH)_2_/graphene

## Abstract

For conventional synthesis of Ni(OH)_2_/graphene hybrids, oxygen-containing functional groups should be firstly introduced on graphene to serve as active sites for the anchoring of Ni(OH)_2_. In this work, a method for growing Ni(OH)_2_ nanosheets on multilayer graphene (MLG) with molecular connection is developed which does not need any pre-activation treatments. Moreover, Ni(OH)_2_ nanosheets can be controlled to stand or lie on the surface of MLG. The prepared hybrids were characterized by X-ray diffraction (XRD), scanning electron microscopy (SEM), transmission electron microscopy (TEM) and X-ray photoelectron spectroscopy (XPS). The growth processes are suggested according to their morphologies at different growth stages. The enhanced electrochemical performances as supercapacitor electrode materials were confirmed by cyclic voltammetry (CV) and galvanostatic charge-discharge (GCD) techniques. Ni(OH)_2_ nanosheets standing and lying on MLG show specific capacities of 204.4 mAh g^−1^ and 131.7 mAh g^−1^, respectively, at 1 A g^−1^ based on the total mass of the hybrids and 81.5% and 92.8% capacity retention at a high current density of 10 A g^−1^, respectively. Hybrid supercapacitors with as-prepared hybrids as cathodes and activated carbon as anode were fabricated and tested.

## 1. Introduction

Supercapacitors recently have gained much attention due to their advantages including large power density, long lifespan, fast charge–discharge rates, free maintenance, and low cost, etc. [1,2,3,4]. Ni(OH)_2_ nanosheets are highly expected to be high-performance electrode materials for supercapacitors due to the large pseudocapacitance [5,6,7,8,9]. However, its low electronic conductivity greatly limits the rate capability and cycling stability [10,11]. Graphene, which has good electronic conductivity, high mechanical strength and large specific surface area [12], is a promising substrate for Ni(OH)_2_ nanosheets to overcome the disadvantage [13,14,15,16,17,18,19,20,21,22,23,24,25,26,27,28,29,30,31,32,33,34,35,36,37].

The morphology and distribution of Ni(OH)_2_ nanosheets in composites have great effect on the electrochemical performances. Compared to a simple physical mixture of Ni(OH)_2_ and graphene, the in situ growth of Ni(OH)_2_ nanosheets on graphene surface will realize much improved performances due to the close contacts and the hierarchical structure of Ni(OH)_2_ nanosheets on graphene. However, the chemical stability of carbon materials and the incompatibility between Ni(OH)_2_ and graphene sheets make it challenging to directly grow Ni(OH)_2_ nanosheets on graphene [15,16]. To overcome this problem, graphene oxide (GO) which provides rich oxygen-containing functional groups on surfaces for preferred nucleation was popularly used as a substrate to synthesize Ni(OH)_2_/graphene [17,18,19]. Various Ni(OH)_2_ nanostructure such as nanoflowers [19], nanoplates [24], nanowall [30], nanosheets [31] and nanoneedles [32] were synthesized on GO based on the electrostatic interaction between nickel ion and the negatively charged GO surface. 

However, GO substrate causes two disadvantages for Ni(OH)_2_/graphene composites. First, oxygen-containing groups and defects on GO will prevent Ni(OH)_2_ nanosheets from growing into large sizes. Secondly, defects and chemical groups remained in reduced graphene oxide decrease the electrical conductivity of composites. Dai groups [24] investigated the effect of various oxidation degrees of graphene on the supercapacitive performances. They found that hexagonal nanoplates were obtained on lightly oxidized graphene with a specific capacitance of 1335 F g^−1^. However, small nanoparticles of Ni(OH)_2_ can be obtained on heavily oxidized graphene and a low specific capacitance of 425 F g^−1^ was then obtained. It demonstrates that electrochemical performances of Ni(OH)_2_/graphene composites can be improved by decreasing the oxidation degree of graphene. Thus, the synthesis of Ni(OH)_2_ nanosheets on graphene without any oxidation are valuable to be investigated.

Here, the direct growth of large Ni(OH)_2_ nanosheets on mechanically exfoliated multilayer graphene (MLG) were realized, which does not need the introduction of oxygen-containing functional groups on graphene at all. Moreover, Ni(OH)_2_ nanosheets could be easily controlled to stand or lie on the surface of MLG. Their microstructures were characterized in detail and electrochemical performances for supercapacitors were tested.

## 2. Experimental Section

### 2.1. Preparation of Samples

NiCl∙6H_2_O, urea, *N*,*N*-dimethylformamide (DMF), KOH, polyvinylidene fluoride and 1-methyl-2-pyrrolidone were purchased from Sinopharm Chemical Reagent Co. Ltd. (Shanghai, China) and used as received. Expanded graphite was purchased from Tianyuanda graphite Co., Ltd. (Qingdao, China).

We added 20 mg expanded graphite into a mixed solvent of DMF and distilled H_2_O with a total volume of 10 mL. After 4 h ultrasonication treatment, the graphite was exfoliated to multilayer graphene (MLG) that was suspended in the solution. Subsequently, a certain amount of NiCl∙6H_2_O and urea were added into the suspension and mixed. The mixture was then poured into a 30 mL of Teflon-lined steel autoclave after homogenous stirring, and it was then sealed and kept at 150 °C for 2 h reaction. After taking out and cooling in the air, the product was washed several times with water and ethanol by centrifugation. The powder was collected after heating at 60 °C for 24 h. S-Ni(OH)_2_/MLG, L-Ni(OH)_2_/MLG, S-Ni(OH)_2_, L-Ni(OH)_2_ were synthesized according to the above procedures by using different masses of raw materials, as listed in Table 1.

### 2.2. Material Characterization

The phases of samples were determined by X-ray diffraction (XRD) which was performed on Shimadzu XRD-6000 (Kyoto, Japan) using Cu Kα radiation (λ = 0.15406 nm) at a scan rate of 4 degree/min. The morphologies and structural properties of the samples were observed on a Hitachi S-4800 field emission scanning electron microscope (FESEM) (Tokyo, Japan) and a FEI SU 8010 transmission electron microscope (TEM) (Hillsboro, OR, USA). The element composition and chemical bonding states of sample were characterized by X-ray photoelectron spectroscopy (XPS, Kratos, AXIS Supra, Manchester, UK).

### 2.3. Electrochemical Measurements

All electrochemical measurements were carried out in a standard three-electrode system with 2 M KOH aqueous solution as electrolyte at room temperature. A Ni foam and an Ag/AgCl electrode were used as the counter and reference electrodes, respectively. The working electrode was prepared by mixing the as-prepared materials, acetylene black and polyvinylidene fluoride at a weight ratio of 80:10:10 and dispersing the mixture in some 1-methyl-2-pyrrolidone to produce homogeneous slurry. After coating the slurry on Ni foam grids (1 cm × 1 cm × 0.2 cm), the electrodes were dried at 80 °C for several hours. The mass loading of as-prepared materials on electrode was about 4 mg cm^−2^. Cyclic voltammetry (CV) curves were performed on a CHI660e electrochemical working station (Shanghai Chenhua Instrument Co., Ltd, Shanghai, China) between 0 and 0.55 V (vs. Ag/AgCl) at different scan rates from 1 to 30 mVs^−1^. The galvanostatic charge-discharge (GCD) curves were measured on a LAND CT2001A test system between 0 and 0.45 V (vs. Ag/AgCl) potential. The specific capacity of electrode was calculated through the GCD curve, and the formula is as follows:

(1) $$Cs=\frac{I\times \Delta t}{3.6\times m}$$

where, *Cs* (mAh g^−1^) is the specific capacity, *I* (A) is the charge and discharge current, Δ*t* (s) is the discharge time, *m* (g) is the mass of electrode active material. Electrochemical impedance spectroscopy (EIS) curves were measured by applying an alternating current with amplitude of 5 mV in the frequency range from 0.01 Hz to 100 kHz.

## 3. Result and Discussion

**Figure 1:**
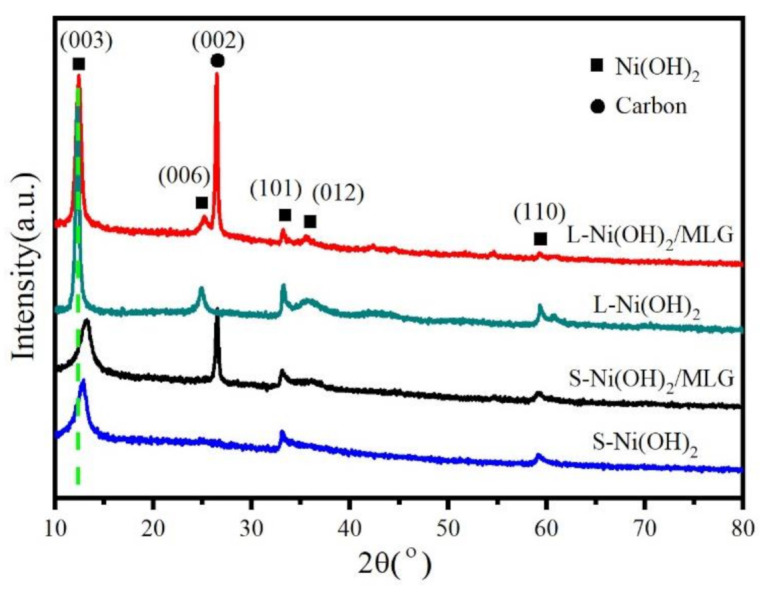
X-ray diffraction (XRD) patterns of samples S-Ni(OH)_2_/MLG, L-Ni(OH)_2_/MLG, S-Ni(OH)_2_ and L-Ni(OH)_2_.

Figure 1 shows the XRD patterns of all samples. The peaks at 26.6° and 53.2° belong to (002) and (004) planes of MLG, respectively (Figure S1). The other peaks at positions of about 12.4°, 25.3° and 33.3° and 59.4° can be well indexed to (003), (006), (100) and (110) planes of Ni(OH)_2_ (Joint Committee on Powder Diffraction Standards (JCPDS) card no. 38-0715). It can be seen that well crystallized Ni(OH)_2_ are present in all samples. Notably, S-Ni(OH)_2_/MLG have rightly shifted (003) peaks when compared to L-Ni(OH)_2_/MLG, indicating the decreased (003) crystal planet distance. This phenomenon may be ascribed to the reduction of interlayer water in Ni(OH)_2_ nanosheets due to high Ni^2+^ concentration and high ratio of DMF/H_2_O in reaction solution. Moreover, S-Ni(OH)_2_/MLG and S-Ni(OH)_2_ have broadened full-width at half-maximum (FWHM) values of (003) peaks and negligible (006) peaks, indicating the ultra-thin thickness of Ni(OH)_2_ nanosheets for S-Ni(OH)_2_/MLG and S-Ni(OH)_2_. 

**Figure 2:**
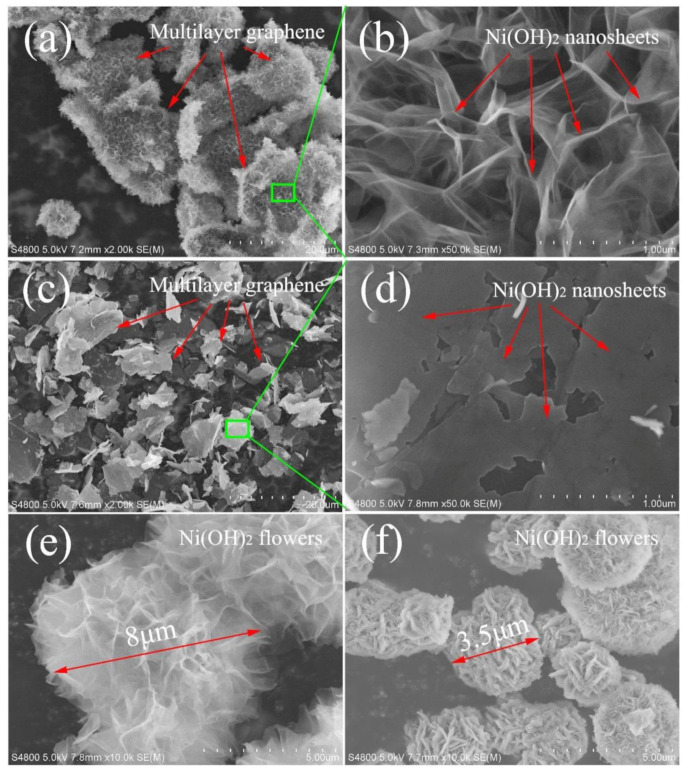
Scanning electron microscope (SEM) images of S-Ni(OH)_2_/MLG ((**a**): low magnification (**b**): high magnification), L-Ni(OH)_2_/MLG ((**c**): low magnification (**d**): high magnification), S-Ni(OH)_2_ (**e**) and L-Ni(OH)_2_ (**f**).

The morphologies of the samples were observed with scanning electron microscopy (SEM). Initial MLG flakes have a flat and smooth surface with a lateral size of several micrometers (Figure S2). The thickness of MLG was measured about 3.5 nm by atomic force microscopy (AFM) [38], meaning MLG consists of 10 to 15 layers. Figure 2a,b show the SEM images of sample S-Ni(OH)_2_/MLG at low and high magnifications, respectively. In Figure 2a, flakes with fluffy surface are obviously observed, indicating MLG sheets are coated with a shell of homogeneous Ni(OH)_2_ layer. When observing Ni(OH)_2_ layers at a high magnification, they are composed of wrinkled Ni(OH)_2_ nanosheets, indicating the ultra-thin property of Ni(OH)_2_ nanosheets. As they are almost vertically standing on MLG with numerous pores in the Ni(OH)_2_ films this makes them a well-organized 3D hierarchical nanostructure. For sample L-Ni(OH)_2_/MLG, the flakes with smooth surface looks like original MLG in a low-magnified SEM image (Figure 2c). However, under a high magnification, Ni(OH)_2_ nanosheets intimately lying on the surface of MLG are observed in Figure 2d. These Ni(OH)_2_ nanosheets have a lateral size above 0.3 μm, which is much larger than previously reported Ni(OH)_2_/GO composite [24]. Moreover, there are only small breaks or even no boundary between the neighbor nanosheets. It indicates that the flat surface and no oxygen-containing function groups facilitate the continuous growth of two-dimensional Ni(OH)_2_ sheets along the surface of MLG.

The morphologies of S-Ni(OH)_2_ and L-Ni(OH)_2_ which were prepared without MLG are shown in Figure 2e,f. Both show flower-like Ni(OH)_2_ flake aggregates. It indicates that MLG can supply as a good substrate for anchoring and growth of Ni(OH)_2_, avoiding the aggregation of Ni(OH)_2_ nanosheets. Sample S-Ni(OH)_2_ shows much thinner Ni(OH)_2_ nanosheets, bigger flower size and fluffier structure than sample L-Ni(OH)_2_. It further demonstrates that the thickness of Ni(OH)_2_ nanosheets is decreased under a high ratio of DMF to H_2_O. However, when further increasing the ratio of DMF/H_2_O to 8:2, less Ni(OH)_2_ is produced and inferior hierarchical structures of Ni(OH)_2_ are obtained on MLG surfaces.

**Figure 3:**
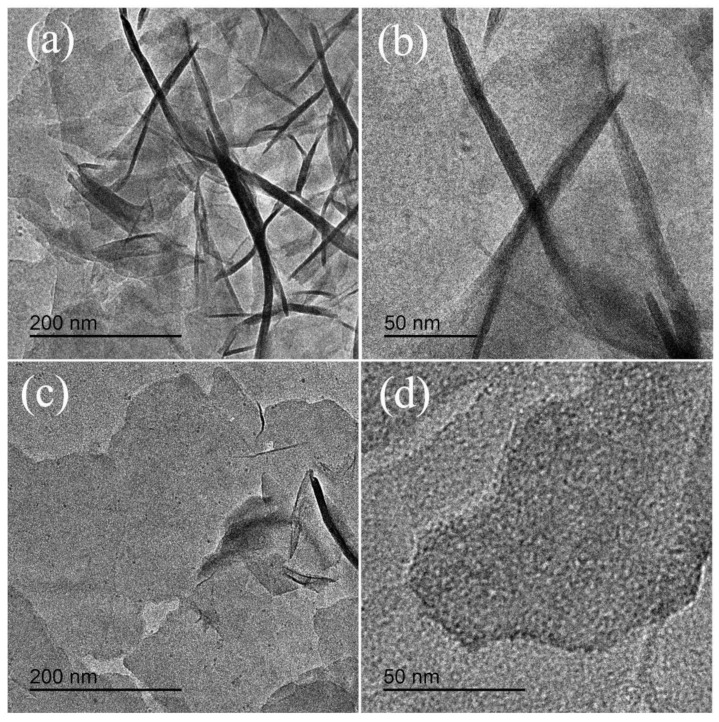
Transmission electron microscopy (TEM) images of S-Ni(OH)_2_/MLG ((**a**): low magnification, (**b**): high magnification) and L-Ni(OH)_2_/MLG ((**c**): low magnification, (**d**): high magnification).

TEM observation was carried out to show detailed information about the structure of S-Ni(OH)_2_/MLG and L-Ni(OH)_2_/MLG, and the images were shown in Figure 3. It can be seen from Figure 3a,b that Ni(OH)_2_ nanosheets with thickness below 5 nm stand on MLG nanosheets randomly in sample S-Ni(OH)_2_/MLG, resulting in many nano-pores between the intersectional Ni(OH)_2_ nanosheets. This porous structure may facilitate the entry of the electrolyte and the ultrathin Ni(OH)_2_ nanosheets can supply more active sites for Faradaic reactions, thus leading to the improvement of electrochemical properties. Conversely, the Ni(OH)_2_ nanosheets intimately lie on MLG in the sample L-Ni(OH)_2_/MLG (Figure 3c,d). The large contact area between Ni(OH)_2_ nanosheets and MLG benefits the electron transfer between them and increases the stability of Ni(OH)_2_ nanosheets during Faradaic reactions, resulting in a high rate performance and long cycling life.

**Figure 4:**
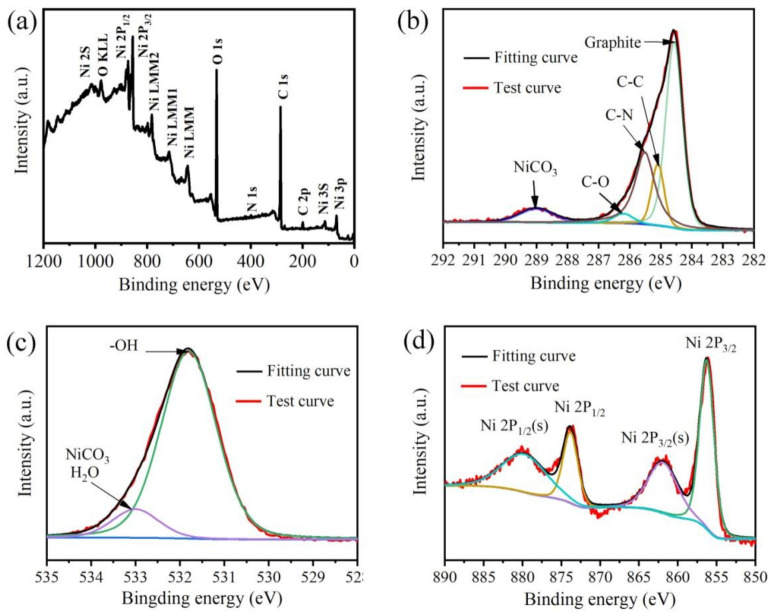
X-ray photoelectron spectroscopy (XPS) spectra of S-Ni(OH)_2_/MLG, (**a**) Survey, (**b**) C 1s, (**c**) O 1s, (**d**) Ni 2p.

XPS was employed to determine the element composition and chemical bonding states of S-Ni(OH)_2_/MLG (Figure 4). The peaks of C, O, Ni and N are observed in the survey spectrum (Figure 4a). High resolution spectra of C 1s, O 1s and Ni 2p are shown in Figure 4b–d. The region of the C1s spectrum can be deconvoluted into five functional groups, which is carbon in graphite, C–C, C–N, C–O and NiCO_3_ (Figure 4b). The C–N may be caused by the interaction between MLG and NH_3_ that comes from the decomposition of urea. The small peak of C–O means that MLG is slightly oxidized, which is also confirmed by the XPS spectrum of pure MLG [39], indicating that Ni(OH)_2_ and MLG cannot be bridged by oxygen functional groups.

The O 1s spectrum shown in Figure 4c is deconvoluted into two peaks at 531.6 eV and 533.1 eV. The peak at 531.6 eV can be attributed to -OH, and another peak at 533.1 eV can be attributed to NiCO_3_ and H_2_O [40,41,42]. The high-resolution Ni 2p spectrum (Figure 4d) is well fitted with a couple of peaks at 856.2 and 873.8 eV, which are ascribed to Ni 2p3/2 and Ni 2p1/2, respectively [43]. Another couple of peaks at 862.0 eV and 880.0 eV are the satellites of Ni^2+^, which indicates the formation of Ni(OH)_2_.

**Figure 5:**
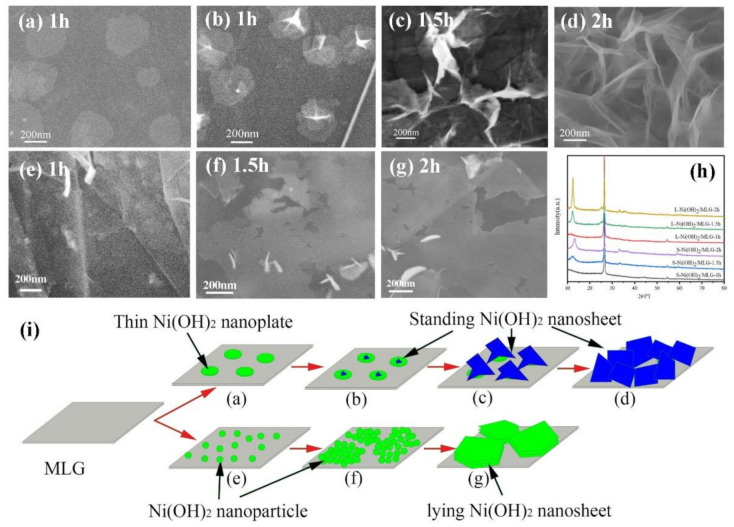
Scanning electron microscope (SEM) images of S-Ni(OH)_2_/MLG (**a**–**d**) and L-Ni(OH)_2_/MLG (**e**–**g**) with different reaction time, and their X-ray diffraction (XRD) patterns (**h**), as well as the suggested schematic growth processes (**i**), Schematic diagrams (a–g) in (i) correspond to SEM images (a–g), respectively.

In order to understand the growth mechanism of Ni(OH)_2_ nanosheets on MLG, S-Ni(OH)_2_/MLG and L-Ni(OH)_2_/MLG with different reaction times of 1 h, 1.5 h and 2 h are prepared and closely observed with SEM. The results are shown in Figure 5a–g. Due to few active functional groups on MLG, the Ni^2+^ ion may be absorbed onto the surface of MLG by van der Waals force after forming complexes with DMF and water, which is similar to our previous synthesis of Co_3_O_4_ nanoparticles on MLG [39]. Then Ni(OH)_2_ are formed and grow under alkaline conditions resulting from urea decomposition. The ratio of DMF to H_2_O is very crucial to the final morphologies of Ni(OH)_2_ nanosheets. When the DMF/H_2_O ratio is 7:3, the evolved Ni(OH)_2_ morphologies are shown in Figure 5a–d. It can be observed from Figure 5a that thin Ni(OH)_2_ nanoplates are formed for 1 h reaction. At the same time, it is also observed that small Ni(OH)_2_ flowers are sprouted out from the center of Ni(OH)_2_ nanoplates (Figure 5b). Then, Ni(OH)_2_ flowers grow larger with the increased reaction time. It can be seen from Figure 5c that adjacent Ni(OH)_2_ flowers will connect to each other for 1.5 h reaction. For 2 h reaction, porous standing Ni(OH)_2_ nanosheets will cover the whole MLG surface (Figure 5d).

With regard to DMF/H_2_O ratio of 2:8, the deposited Ni(OH)_2_ is nanoparticles on MLG surface for 1 h (Figure 5e). The gradual deposition will increase the density, thickness and crystallization of Ni(OH)_2_, forming of dense Ni(OH)_2_ nanoparticle cluster on MLG (Figure 5f). Finally, the Ni(OH)_2_ clusters are converted to thick, large and crystallized Ni(OH)_2_ nanosheets which intimately lie on MLG for 2 h reaction (Figure 5g). Figure 5h shows the XRD patterns of the samples with different reaction time. It is observed that all the diffraction peaks of Ni(OH)_2_ become stronger with the increased reaction time, indicating the increased Ni(OH)_2_ content in composites. Thus, the growth processes of Ni(OH)_2_ on MLG under different DMF/H_2_O ratios are schematically demonstrated in Figure 5i.

Cyclic voltammetry (CV) measurement was carried out to investigate the electrochemical processes of the samples. Figure 5a,b show the CV curves of S-Ni(OH)_2_/MLG and L-Ni(OH)_2_/MLG at the scan rates of 1, 5, 10, 20 and 30 mV s^−1^ within a potential range from 0 to 0.55 V, respectively. One pair of redox peaks can be clearly observed, resulting from the Faradic reactions of Ni(OH)_2_ in alkaline electrolyte, which can be described as the following equation:

Ni(OH)_2_ + OH^−^ ↔ NiOOH + H_2_O + e^−^

When comparing the CV curves of S-Ni(OH)_2_/MLG and L-Ni(OH)_2_/MLG at the same scan rate of 5 mV s^−1^, it can be seen that the integrated area of the enclosed CV curve of S-Ni(OH)_2_/MLG is much larger than that of L-Ni(OH)_2_/MLG, which suggests the larger specific capacity of S-Ni(OH)_2_/MLG than that of L-Ni(OH)_2_/MLG. It ascribes to the porous standing nanostructures and its relative higher content of Ni(OH)_2_ in the composite. Meanwhile, the redox peaks of L-Ni(OH)_2_/MLG are not as sharp as that of S-Ni(OH)_2_/MLG. It may be caused by its thicker Ni(OH)_2_ nanosheets, which will decrease the electrochemical reactivity.

The b value can be estimated from the power law equation *I* = a*v*^b^ to determine the energy storage mechanism, where *i* corresponds to anode or cathode peak current of the CV curve, *v* is the scan rate, a and b are variable parameters. S-Ni(OH)_2_/MLG and L-Ni(OH)_2_/MLG have b values of 0.58 and 0.52 respectively, by calculating from the slops of log(*i*) vs. log(*v*) plots (Figures S3 and S4). The results indicate that both of them exhibit dominant Faradaic (battery-type) behavior.

**Figure 6:**
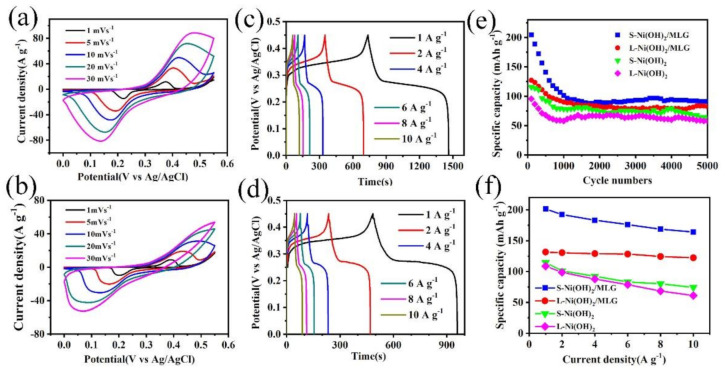
Electrochemical performances of electrodes, cyclic voltammetry (CV) curves of S-Ni(OH)_2_/MLG (**a**) and L-Ni(OH)_2_/MLG (**b**) from a scan rate of 1–30 mV s^−1^, galvanostatic charge-discharge (GCD) curves of S-Ni(OH)_2_/MLG (**c**) and L-Ni(OH)_2_/MLG (**d**) at a current density of 1–10 A g^−1^, (**e**) Specific capacities at various current densities. (**f**) Cycling performance at a current density of 4 A g^−1^.

Figure 6c,d show the GCD curves of S-Ni(OH)_2_/MLG and L-Ni(OH)_2_/MLG under different current densities. Both samples show very obvious charge/discharge plateaus at low current density, indicating a pseudocapacitive feature of electrodes from redox reactions of Ni(OH)_2_. At high current densities, the plateaus are still distinguished, indicating the fast redox reaction process. Meanwhile, the charge and discharge plateaus are not obviously positively and negatively shifted, indicating a small polarization produced in electrodes at high current densities. The specific capacities calculated from the discharge curves at different current densities are shown in Figure 6f. A specific capacity of 204.4 mAh g^−1^ is delivered at 1 A g^−1^ for sample S-Ni(OH)_2_/MLG based on the total mass of Ni(OH)_2_ and MLG. With the increase of current density, the capacity gradually decreases to 81.5% retention at 10 A g^−1^. As a comparison, L-Ni(OH)_2_/MLG delivers a specific capacity of 131.7 mAh g^−1^ at 1 A g^−1^ and 92.8% capacity retention at 10 A g^−1^. The comparison of the as-prepared Ni(OH)_2_/MLG hybrid with the previously reported Ni(OH)_2_ based hybrids was briefly summarized in Table S1. By comparison, the S-Ni(OH)_2_/MLG prepared in this work is at a high level of specific capacity with high active material loading. It is noteworthy that the mass ratios of Ni(OH)_2_ in S-Ni(OH)_2_/MLG and L-Ni(OH)_2_/MLG are calculated to be about 71.4% and 45.9% respectively by weighing the MLG before and after the chemical deposition process. It demonstrates that Ni(OH)_2_ in both composites almost reaches its theoretical capacity (289 mAh g^−1^), which can be ascribed to the ultra-thin properties and homogeneous distribution on MLG. The relative higher rate performance of L-Ni(OH)_2_/MLG may be attributed to the larger contact area between Ni(OH)_2_ and MLG and a lower content of Ni(OH)_2_ in composite. S-Ni(OH)_2_ and L-Ni(OH)_2_ exhibit lower specific capacity and rate performance than S-Ni(OH)_2_/MLG and L-Ni(OH)_2_/MLG, respectively, indicating that the electrochemical performance of Ni(OH)_2_ can be greatly improved by the MLG substrate due to the introduction of conductive networks.

Cycling stability was tested at the current density of 4 A g^−1^, and the results are shown in Figure 6e. Specific capacities of 91.1 mAh g^−1^ and 82.2 mAh g^−1^ are maintained after 5000 cycles for S-Ni(OH)_2_/MLG and L-Ni(OH)_2_/MLG electrodes, showing 45.2% and 64.8% capacity retention, respectively. The decreased specific capacities may be related to the detachment and dissolution of Ni(OH)_2_ from electrodes due to the volumetric changes induced by redox reaction during the cycling, leading to the loss of the electrochemical activity. Improved cycling stability of L-Ni(OH)_2_/MLG may be related to the increased contact surfaces between Ni(OH)_2_ and MLG. S-Ni(OH)_2_ and L-Ni(OH)_2_ electrodes show the specific capacities of 62.2 mAh g^−1^ and 57.8 mAh g^−1^ after 5000 cycles, which is much lower than those of S-Ni(OH)_2_/MLG and L-Ni(OH)_2_/MLG electrodes. The electrochemical performance of MLG is presented in Figures S5–S7. It can be seen that MLG shows a typical electric double layer capacitive behavior with rectangular CV curve and triangular GCD curve. MLG shows low specific capacity about 2.67 mAh g^−1^ (12 F g^−1^) and high stability without fading after 5000 cycles at a current density of 2 A g^−1^. It indicates the high performances of S-Ni(OH)_2_/MLG and L-Ni(OH)_2_/MLG hybrids may be attributed to the synergistic effect between Ni(OH)_2_ and MLG.

**Figure 7:**
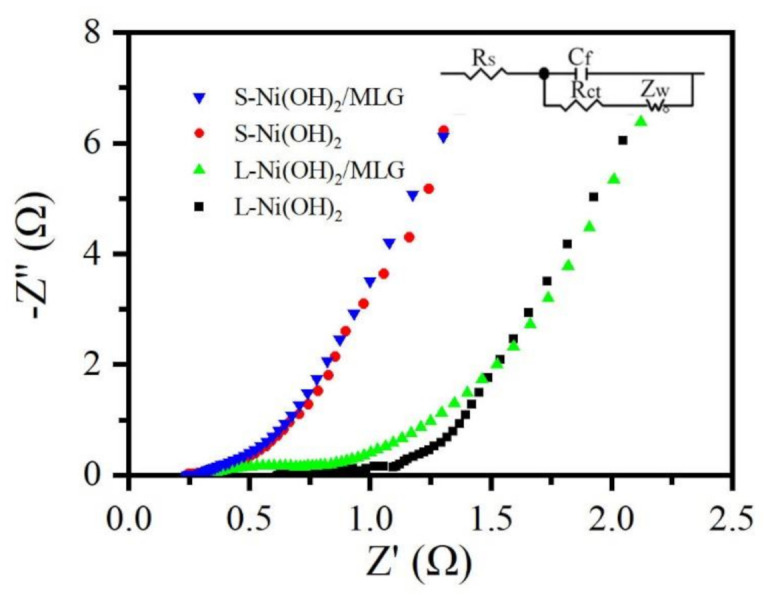
Nyquist plots of S-Ni(OH)_2_/MLG, L-Ni(OH)_2_/MLG, S-Ni(OH)_2_ and L-Ni(OH)_2_, the inset shows the equivalent circuit diagram.

The electrochemical impedance spectroscopy (EIS) curves of S-Ni(OH)_2_/MLG, L-Ni(OH)_2_/MLG, S-Ni(OH)_2_ and L-Ni(OH)_2_ were measured. Their Nyquist plots are shown in Figure 7. An equivalent circuit shown in the inset of Figure 7 was used to fit the Nyquist plots. Rs represents the solution resistance. Rct represents the electrochemical reaction impedance. Z_W_ is Warburg diffusion impedance, which reflects the diffusion information of the capacitor and indicates the diffusion resistance of the capacitor. C_f_ is the interface capacitance. The impedance data obtained by fitting are listed in Table S2. It can be seen that Rs values of S-Ni(OH)_2_/MLG and L-Ni(OH)_2_/MLG are 0.23 Ω and 0.3 Ω, respectively, which is smaller than that of S-Ni(OH)_2_ and L-Ni(OH)_2,_ respectively. This indicates that electronic movements in electrodes are improved due to high conductive MLG substrate and well constructed nanostructures of Ni(OH)_2_ and MLG. It also can be seen that Rct values of S-Ni(OH)_2_/MLG and S-Ni(OH)_2_ are 0.08 Ω, which is much smaller than that of L-Ni(OH)_2_/MLG (0.4 Ω) and L-Ni(OH)_2_ (0.4 Ω). It may due to the thinner thickness of Ni(OH)_2_ nanosheets in S-Ni(OH)_2_/MLG and S-Ni(OH)_2_, which improves the redox activity of Ni(OH)_2_. The slopes of the straight lines in the low frequency region are steep for all samples, reflecting that all electrodes have low ion diffusion resistance.

**Figure 8:**
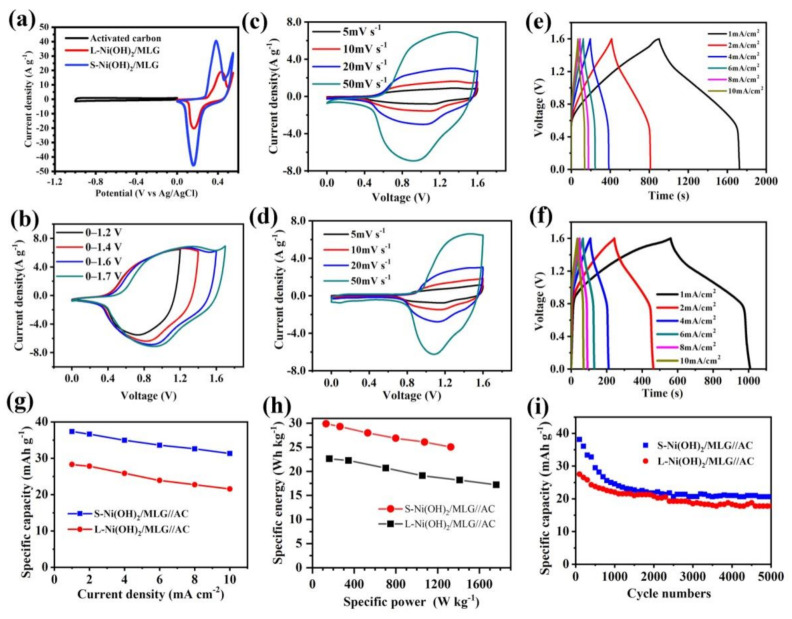
CV curves of S-Ni(OH)_2_/MLG, L-Ni(OH)_2_/MLG and activated carbon (AC) electrodes at a scan rate of 10 mV s^−1^ (**a**), CV curves of S-Ni(OH)_2_/MLG//AC with different voltage scan ranges (**b**), CV curves of S-Ni(OH)_2_/MLG//AC (**c**) and L-Ni(OH)_2_/MLG//AC (**d**) at different scan rates, GCD curves of S-Ni(OH)_2_/MLG//AC (**e**) and L-Ni(OH)_2_/MLG//AC (**f**), Rate performance (**g**), Ragone plot (**h**) and cycling performance (**i**) of S-Ni(OH)_2_/MLG//AC and L-Ni(OH)_2_/MLG//AC.

In order to further evaluate the electrochemical performances, hybrid supercapacitors using S-Ni(OH)_2_/MLG or L-Ni(OH)_2_/MLG as cathode, activated carbon (AC) as anode and filter paper as a separator were assembled. The loadings of active materials in cathodes and anodes were controlled to meet the charge balance m_c_F_c_ΔV_c_ = m_a_F_a_ΔV_a_, where m is the active mass, F is the specific capacity, ΔV is the potential window, subscript c is for cathode and subscript a is for anode. Accordingly, the mass of Ni(OH)_2_/MLG and activated carbon loaded on electrodes were controlled to be about 2 mg cm^−2^ and 8 mg cm^−2^ for S-Ni(OH)_2_/MLG//AC, and about 2.7 mg cm^−2^ and 8 mg cm^−2^ for L-Ni(OH)_2_/MLG//AC, respectively. The electrochemical performances of hybrid supercapacitors were tested in 2 M KOH solution in a two-electrode system. Figure 8a shows the CV curves of S-Ni(OH)_2_/MLG, L-Ni(OH)_2_/MLG and activated carbon in the three-electrode system alone. S-Ni(OH)_2_/MLG and L-Ni(OH)_2_/MLG have a strong redox process, indicating a good pseudo-capacitive effect. However, the activated carbon electrode displays a typical rectangle shape, indicating typical EDLC behavior. The hybrid supercapacitors were assembled to combine the individual advantages to realize a high energy density under a power density.

CV curves with different voltage scan ranges for the S-Ni(OH)_2_/MLG//AC capacitor were obtained at the scan rate of 50 mVs^−1^ and shown in Figure 8b. The results show that the hybrid supercapacitor undergoes the similar redox processes under different voltage scan ranges. It indicates the high reversibility of the redox reactions. Different scan rates at 5, 10, 20, 50 mV s^−1^ were applied on the capacitors of S-Ni(OH)_2_/MLG//AC and L-Ni(OH)_2_/MLG//AC, and the results are shown in Figure 8c,d, respectively. The redox peak currents increase with the increased scan rates, indicating the redox reactions can respond well to the high scan rates. S-Ni(OH)_2_/MLG//AC show a higher anode and cathode current and wider potential range when compared to L-Ni(OH)_2_/MLG//AC. It may be attributed to its 3D nanostructure and ultrathin Ni(OH)_2_ nanosheets of S-Ni(OH)_2_/MLG, which can provide high EDLC current and higher electrochemical redox activity.

GCD curves of capacitors of S-Ni(OH)_2_/MLG//AC and L-Ni(OH)_2_/MLG//AC at various current densities are shown in Figure 8e,f, respectively. The charge and discharge curves are mostly symmetric, indicating the high reversibility and high columbic efficiency. The charge and discharge curves have similar shape at various current densities, meaning good rate capability. The capacities of two capacitors at different currents are shown in Figure 8g. S-Ni(OH)_2_/MLG//AC and L-Ni(OH)_2_/MLG//AC exhibit 37.4 mAh g^−1^ and 28.3 mAh g^−1^ at 1 mA cm^−^^2^, and 84% and 76% capacity retention at 10 mA cm^−1^, respectively, based on the total mass of active materials in cathode and anode electrodes, showing a superior rate performance. The unsatisfied energy density may be caused by the lower capacitance of activated carbon, which has a specific capacitance about 180 F/g (50 mAh g^−1^) under the potential window from −1.0 V to 0 V. As shown in Figure 8h, Ragone plots of S-Ni(OH)_2_/MLG//AC and L-Ni(OH)_2_/MLG//AC are calculated from their rate performances. S-Ni(OH)_2_/MLG//AC delivers a high specific energy of 30 Wh kg^−1^ at 130 W kg^−1^ and 25 Wh kg^−1^ at a high specific power of 1326 W kg^−1^. Meanwhile, L-Ni(OH)_2_/MLG//AC delivers 22 Whkg^−1^ at 160 W kg^−1^ and 17.2 Whkg^−1^ at 1761 W kg^−1^, a little lower than those of S-Ni(OH)_2_/MLG//AC.

Cycling performance of S-Ni(OH)_2_/MLG//AC and L-Ni(OH)_2_/MLG//AC were measured at a current density of 4 mA cm^−2^ (Figure 8i). With the increase of cycle numbers, the specific capacities are gradually decreased. The retention of specific capacity of 54% and 66% are obtained for S-Ni(OH)_2_/MLG//AC and L-Ni(OH)_2_/MLG//AC, respectively, after 5000 cycles. These results show that both of them exhibit high performances as supercapacitor anode materials.

## 4. Conclusions

In summary, a chemical method based on van der Waals force interaction was developed to directly synthesize Ni(OH)_2_ nanosheets on MLG with different statues. Benefiting from the smooth surface and without the introduction of activated functional groups on the MLG surface, Ni(OH)_2_ nanosheets can be homogeneously grown on the MLG. Two typical morphologies, a porous standing Ni(OH)_2_ nanosheet and an intimate lying Ni(OH)_2_ nanosheet, were synthesized on MLG by controlling the synthesis conditions and the volumetric ratio of DMF to water. The growth processes of Ni(OH)_2_ nanosheets on MLG are well studied. Due to the different mass contents and arrangement, the hierarchical nanostructure of porous standing Ni(OH)_2_ nanosheet/MLG enable them to exhibit a high specific capacity. The intimate contact of lying Ni(OH)_2_ nanosheet/MLGs enables them to have an excellent rate capability.

## Figures and Tables

**Table 1:** Masses of raw materials to synthesize different samples.

Sample Name	Expanded Graphite	DMF/H_2_O Ratio	NiCl·6H_2_O	Urea
S-Ni(OH)_2_/MLG	20 mg	7:3	170 mg	70 mg
L-Ni(OH)_2_/MLG	20 mg	2:8	50 mg	40 mg
S-Ni(OH)_2_	0 mg	7:3	170 mg	70 mg
L-Ni(OH)_2_	0 mg	2:8	50 mg	40 mg

## Data Availability

Data is contained within the article or Supplementary Material.

## Supplementary Materials

The following are available online at https://www.mdpi.com/article/10.3390/nano11071662/s1, Figure S1: XRD pattern of MLG, Figure S2: SEM images of pure MLG, Figure S3: Log(*i*) vs. log(*v*) plots of S-Ni(OH)_2_/MLG, Figure S4: Log(*i*) vs. log(*v*) plots of L-Ni(OH)_2_/MLG, Figure S5: CV curves of MLG at different scan rates, Figure S6: GCD curves of MLG at different current densities, Figure S7: Cycling performance of MLG at a current density of 2 A g^−1^, Table S1: The electrochemical performances of reported Ni(OH)_2_-based hybrids and as-prepared S-Ni(OH)_2_/MLG, Table S2: Impedance data of S-Ni(OH)_2_/MLG, L-Ni(OH)_2_/MLG, S-Ni(OH)_2_ and L-Ni(OH)_2_ electrodes.
