# Supplementary material for: Standing and Lying Ni(OH)2 Nanosheets on Multilayer Graphene for High-Performance Supercapacitors
Source: Nanomaterials (Basel). 2021 Jun 24;11(7):1662. doi: 10.3390/nano11071662 (PMC8308107; doi:10.3390/nano11071662)
Supplement: Supplementary file 1 [file nanomaterials-11-01662-s001.zip › nanomaterials-1265348-supplementary.pdf]

Standing and lying $Ni(OH)_2$ nanosheets on multilayer graphene for high performance supercapacitors

Junming Xu[1*], Mengxia Tang[1], Zhengming Hu[1], Xiaoping Hu[1], Tao Zhou[1], Kaixin Song[1], Jun Wu[1], Jipeng Cheng[2,3,*]

1 College of Electronic Information, Hangzhou Dianzi University, Hangzhou 310018, PR China
2 School of Materials Science & Engineering, Zhejiang University, Hangzhou 310027, PR China
3 School of Physics and Microelectronics, Zhengzhou University, Zhengzhou 450052, China
* Corresponding author: xujunming@hdu.edu.cn, chengjp@zju.edu.cn

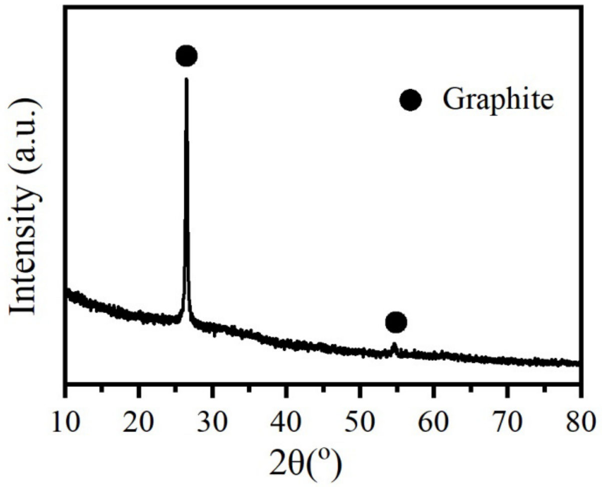

Figure S1 XRD pattern of MLG

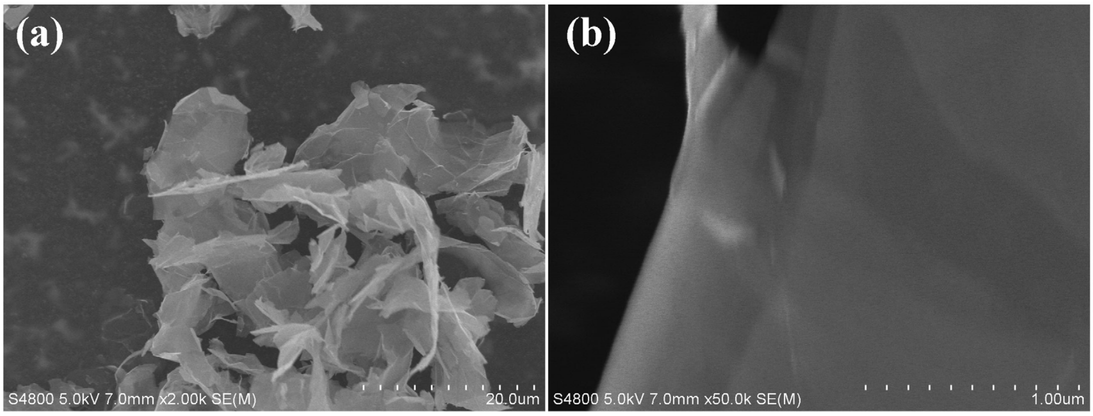

Figure S2 SEM images of pure MLG (a: low magnification, b: high magnification)

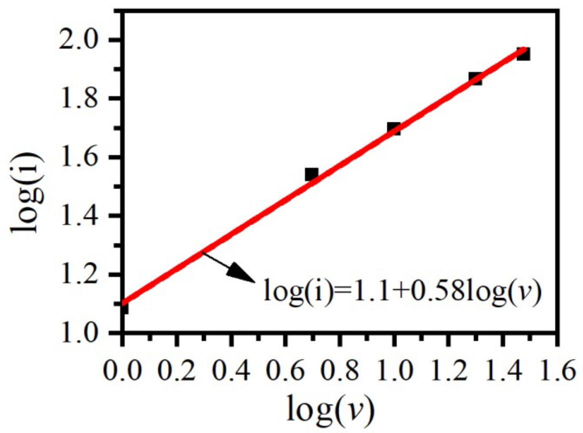

Figure S3 Log(i) vs. log(v) plots of S-$Ni(OH)_2$/MLG

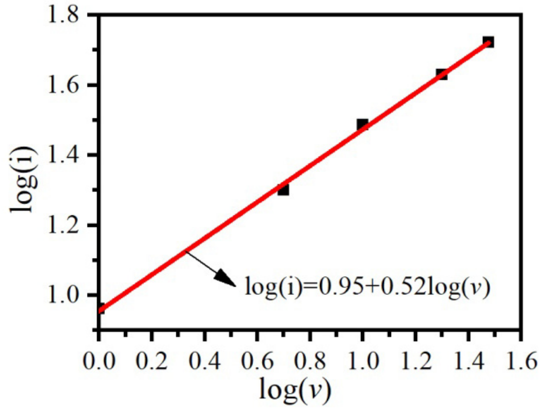

Figure S4 Log(i) vs. log(v) plots of L-$Ni(OH)_2$/MLG

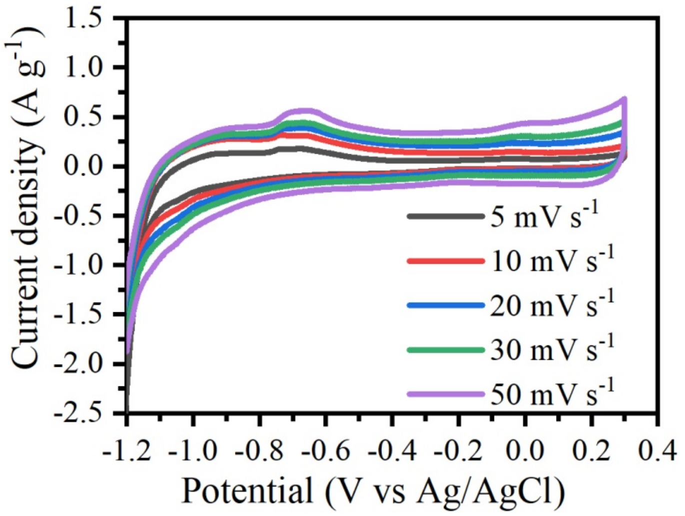

Figure S5 CV curves of MLG at different scan rates

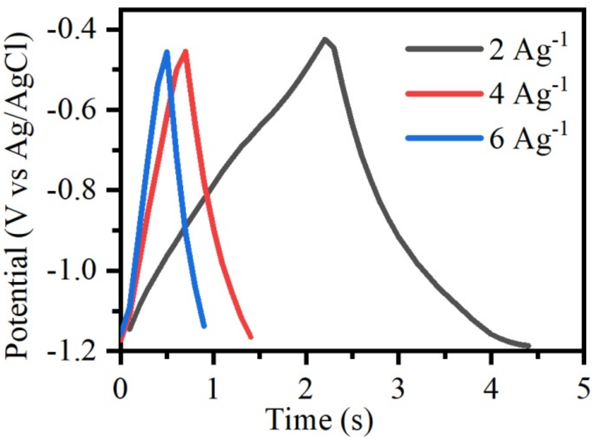

Figure S6 GCD curves of MLG at different current densities

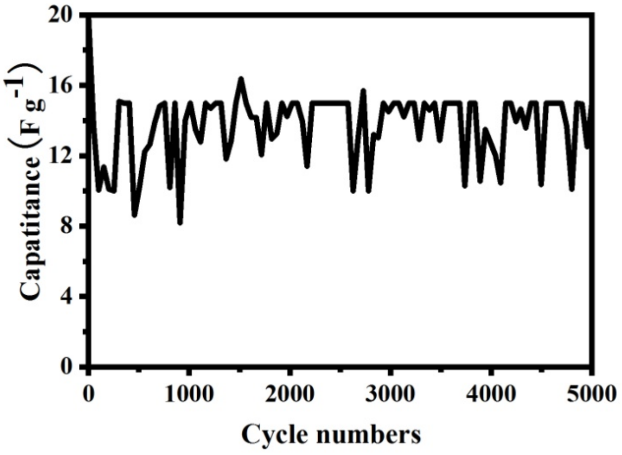

Figure S7 Cycling performance of MLG at a current density of 2 A g^{-1}

Table S1 The electrochemical performances of reported $Ni(OH)_2$-based hybrids and as-prepared S-$Ni(OH)_2$/MLG

$Ni(OH)_2$ based hybrids	Electrolyte	Discharge current (A g^{-1})	Load(mg cm^{-2})	Potential window(V)	Specific Capacity (mAh g^{-1})	Ref
Carbon nanotubes/graphene-doped $Ni(OH)_2$ thin film	1 M KOH	5	1.92	0−0.5 V	257.7	[13]
$Ni(OH)_2$ coating on carbon nanosheets	6 M KOH	1	1	0−0.5 V	308	[16]
Ultrathin $Ni(OH)_2$ layer coupling with graphene	6 M KOH	1	0.5	0−0.5 V	217	[18]
$Ni(OH)_2$-graphene sheet-carbon nanotube composite	6 M KOH	0.2	8	0−0.55 V	178.8	[20]
Layered Inorganic-Organic Hybrid Material Based on Reduced Graphene Oxide and α-$Ni(OH)_2$	1 M KOH	1	1	0−0.6 V	278.6	[22]
Layer-by-layer inkjet printing GO film anchored $Ni(OH)_2$ nanoflakes	3 M KOH	1	3.7	0−0.5 V	192.5	[26]
$Ni(OH)_2$/g-C_3N_4/RGO	6 M KOH	1	-	0−0.53 V	80	[27]
$Ni(OH)_2$ nanoneedles on N-doped 3D rivet graphene fifilm	6 M KOH	1	3.3	0−0.5V	256.1	[32]
$Ni(OH)_2$-reduced graphene oxide composite	6 M KOH	1	-	0−0.45 V	78.4	[34]
Free-standing N-Graphene as conductive matrix for $Ni(OH)_2$	2 M KOH	1	-	-0.2−0.4 V	107	[35]
Al-doped a-$Ni(OH)_2$/reduced graphene oxide composite	6 M KOH	1	2.0	0−0.53 V	376.6	[36]
S-$Ni(OH)_2$/multilayer graphene	2 M KOH	1	4.0	0−0.45 V	204.4	Our work

Table S2 Impedance data of S-$Ni(OH)_2$/MLG, L-$Ni(OH)_2$/MLG, S-$Ni(OH)_2$ and L-$Ni(OH)_2$ electrodes

electrode	Rs(Ω)	Rct(Ω)	Cf(μF)	Zw(Ω)
S-$Ni(OH)_2$/MLG	0.23	0.08	3.21	2.46
S-$Ni(OH)_2$	0.25	0.08	2.81	2.74
L-$Ni(OH)_2$/MLG	0.3	0.4	0.13	2.34
L-$Ni(OH)_2$	0.6	0.4	0.02	2.04
